# Use of High Condylectomy and Abdominal Dermis Fat Grafting for an Acromegaly Induced Bilateral Condylar Hyperplasia: A Case Report

**DOI:** 10.7759/cureus.47039

**Published:** 2023-10-14

**Authors:** Charudatta S Naik, Sanjay S Joshi, Bhupendra V Mhatre, Aarti R Garad, Rushika M Jain

**Affiliations:** 1 Department of Oral and Maxillofacial Surgery, Terna Dental College, Navi Mumbai, IND

**Keywords:** high condylectomy, tmj pain, abdominal dermis fat, condylar hyperlasia, acromegaly complications

## Abstract

Acromegaly is a disorder characterized by hypersecretion of growth hormone, resulting in morbidities associated with multiple systems. Although most of the morbidities are reversed following control of the underlying disease, it may take several weeks to months for the symptoms to subside. One of the most noticeable effects of acromegaly is changes in facial features and jawbone growth, which can lead to severe pain and discomfort. This report describes a case of a 31-year-old patient with acromegaly induced bilateral condylar hyperplasia who presented with severe temporomandibular joint (TMJ), facial pain, and degenerative changes in TMJ. The patient was treated by trans-sphenoidal excision of pituitary adenoma, medications, and radiotherapy, but his hormonal levels were persistently high. Considering the refractory nature of the disease, the patient underwent bilateral high condylectomy, right articular disc removal, and abdominal dermis fat grafting. The surgery arrested the progressive mandibular enlargement and prevented further degenerative changes of TMJ. Although there was some reduction in TMJ pain, the myogenous pain and headache persisted after surgery. TMJ surgery may be selectively used for refractory cases of acromegaly and those requiring discectomy or total joint replacement. This case report describes the role of TMJ surgery in the management of morbidities and symptoms associated with TMJ in acromegaly until biochemical normalcy is achieved.

## Introduction

Acromegaly is a disorder characterized by hypersecretion of growth hormone (GH), resulting in morbidities associated with multiple systems [1]. The most common cause of acromegaly is pituitary adenoma [2]. Although the term acromegaly describes the enlargement of limbs, there are several morbidities associated with this disorder, such as diabetes mellitus, hypertension, headache, arthralgias, dental and facial changes, obstructive sleep apnea, intestinal polyps, visual impairment, excessive sweating, fatigue, the possibility of developing malignancies, altered sleep patterns, and psychosocial problems.

The treatment for pituitary adenoma-induced acromegaly is the surgical removal of the tumor and medical management and/or radiation therapy to treat the residual disease, if any. The primary goal of this treatment is to achieve normalcy of age-normalized serum insulin-like growth factor-1 (IGF-1) value, which signifies control of acromegaly. Even with the most favorable outcome, this may take a variable amount of time, ranging from weeks to months [1]. Patients continue to suffer from their morbidities till hormonal stabilization is achieved. In many cases, even after stabilization of IGF-1 and GH levels, some symptoms, such as arthralgia, headache, and facial myalgia, may persist. Most of the morbidities mentioned above are reversed following successful treatment of the tumor except for skeletal and dental changes, which require further management after normalization of IGF-1 and GH levels [2].

This report describes a case of acromegaly induced bilateral condylar hyperplasia with internal derangement of the temporomandibular joint (TMJ), which was managed by high condylectomy and abdominal dermis fat grafting. The impetus of this case report is to discuss the management of morbidities and symptoms associated with TMJ till hormonal levels are stabilized.

## Case presentation

**Figure 1:**
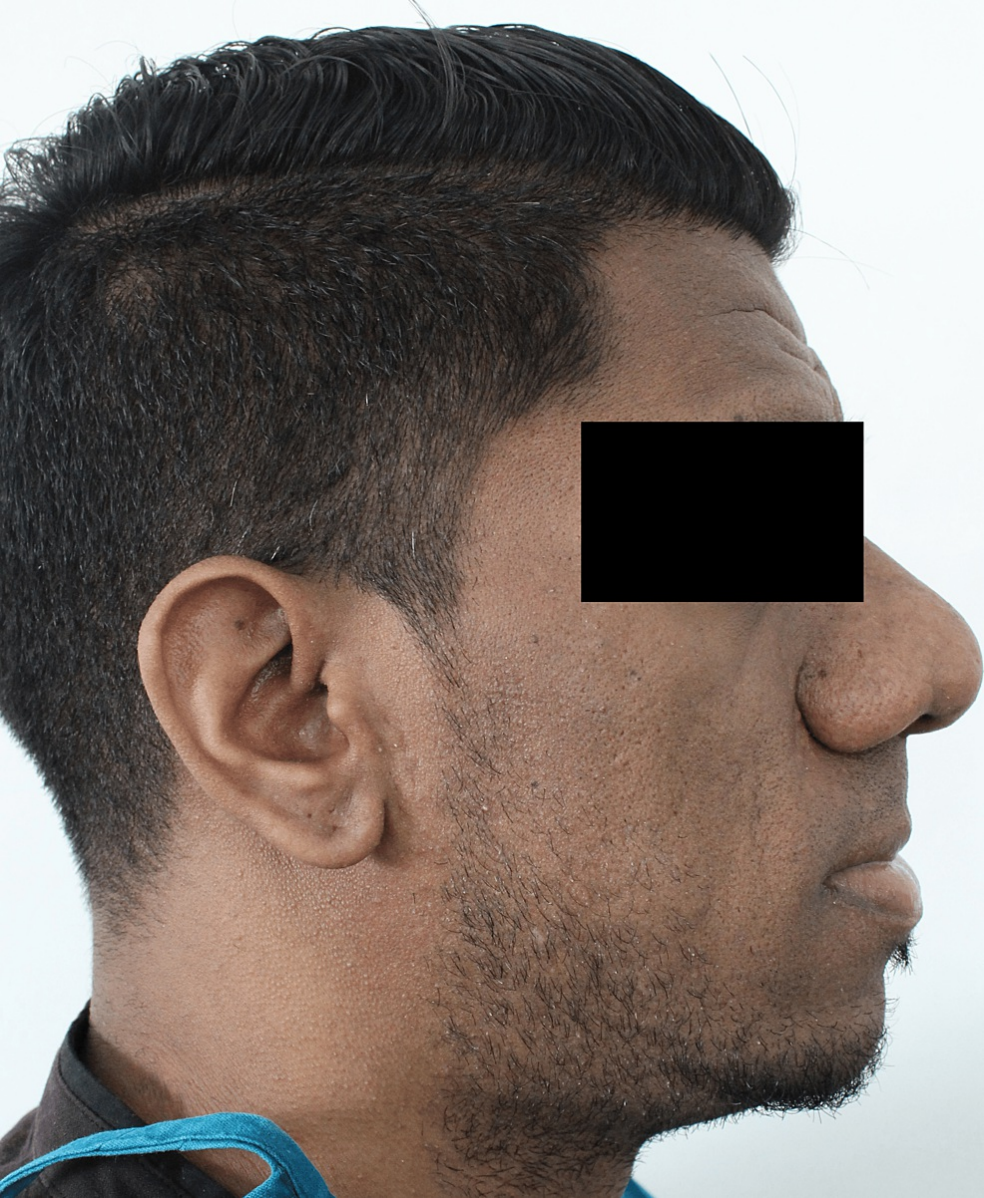
Profile view showing prognathic mandible

A 31-year-old male reported to us with a complaint of bilateral preauricular and facial pain and progressive enlargement of the lower jaw (Figure 1).

The patient also complained of headaches intermittently. His medical history revealed that he was diagnosed with acromegaly secondary to pituitary macroadenoma. The patient had undergone trans-sphenoidal excision of pituitary macroadenoma one year back. There was a residual tumor in the pituitary, which was to be managed by medicinal therapy and radiation. His GH and IGF-1 levels were persistently high following surgery and medicinal therapy, which included cabergoline. Table 1 shows his IGF-1 and GH levels at various times during his treatment.

**Table 1:** Treatment timeline and corresponding IGF-1 and GH values GH, growth hormone; IGF-1, insulin-like growth factor; TMJ, temporomandibular joint

	IGF-1 (somatomedin C) levels	GH levels
Preoperative (before excision of adenoma)	747 ng/mL	38 ng/mL
Trans-sphenoidal excision of pituitary macroadenoma done		
6 months post-adenoma excision	613 ng/mL	-
Started on medications like cabergoline		
13 months post-pituitary surgery	TMJ surgery done	
16 months post-pituitary surgery	675 ng/mL	24.8 ng/mL
Radiation therapy started		
1-year post-radiation therapy	541 ng/mL	18.9 ng/ mL
Started on injection octreotide		

**Figure 2:**
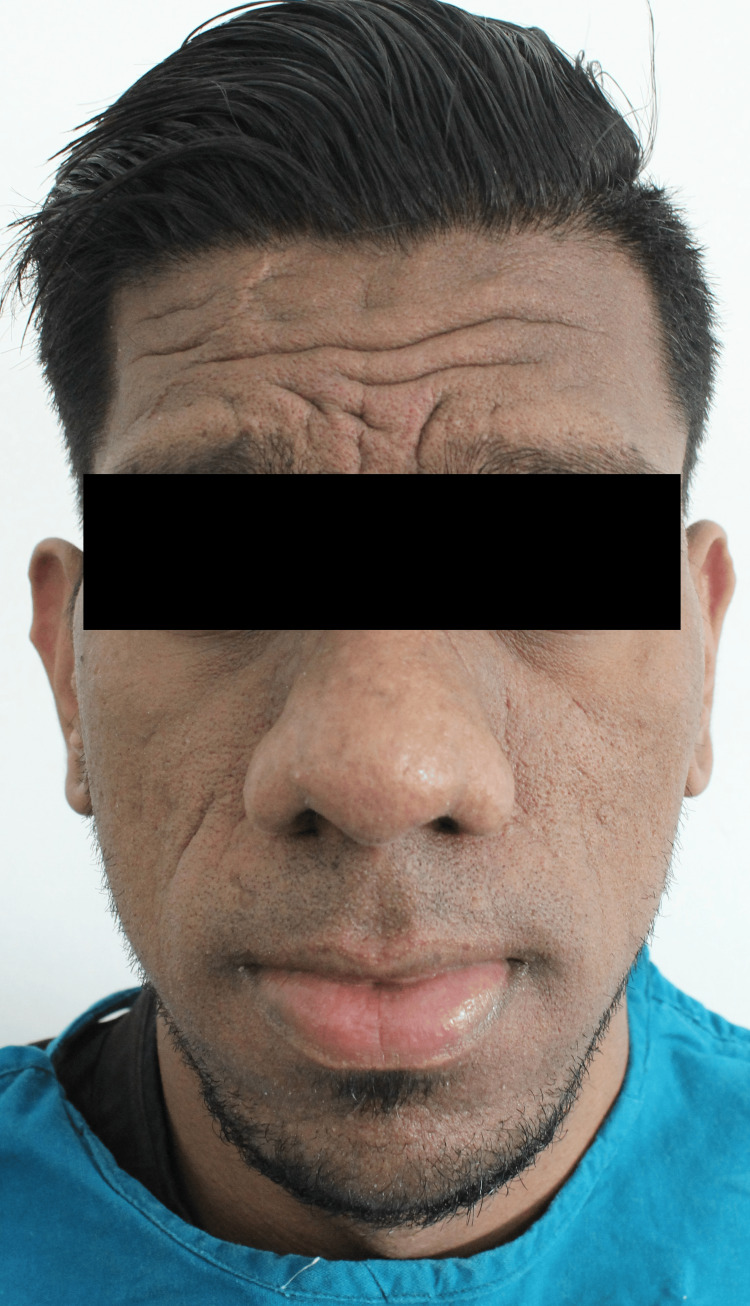
Frontal view showing characteristic features of acromegaly The figure shows frontal bossing, prognathic mandible, thick everted lower lip, broad nose, and coarse facial skin

Clinical examination revealed frontal bossing, prognathic mandible, thick everted lower lip, broad nose, and coarse facial skin (Figure 2).

**Figure 3:**
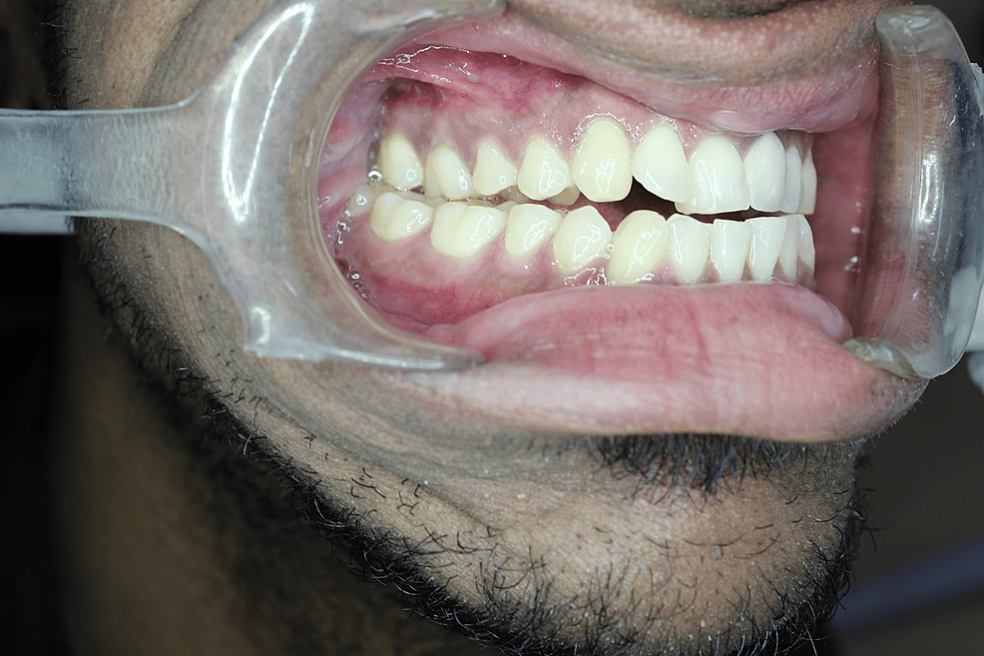
Intraoral view showing class III dental malocclusion

There was severe tenderness bilaterally over the TMJ region. He had pain and clicking during mouth opening, although mouth opening was adequate. Intraoral examination showed macroglossia, such as class III dental malocclusion, which was worsening (Figure 3).

**Figure 4:**
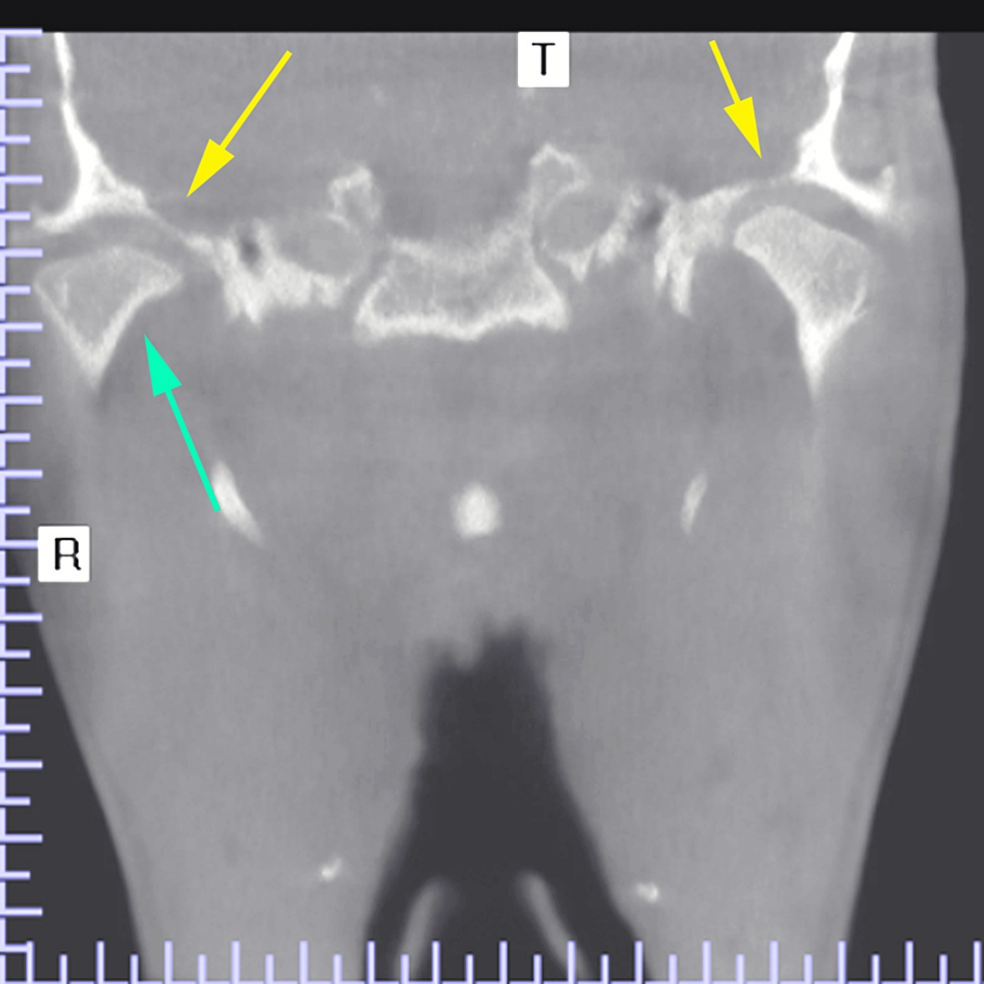
CBCT coronal section showing erosion of the right condyle (green arrow) and thinning of the roof of the glenoid fossa (yellow arrow) CBCT, cone beam computed tomography

The patient gave a history of persistent enlargement of the lower jaw, hands, and feet. An orthopantomogram and a cone beam computed tomography (CBCT) were advised, which showed reduced joint spaces bilaterally, erosion of the right condyle, and thinning of the roof of the glenoid fossae (Figure 4).

TMJ magnetic resonance imaging (MRI) revealed anterior disc displacement with reduction (ADDR) and thinning of the articular disc, which was more prominent on the right side.

**Figure 5:**
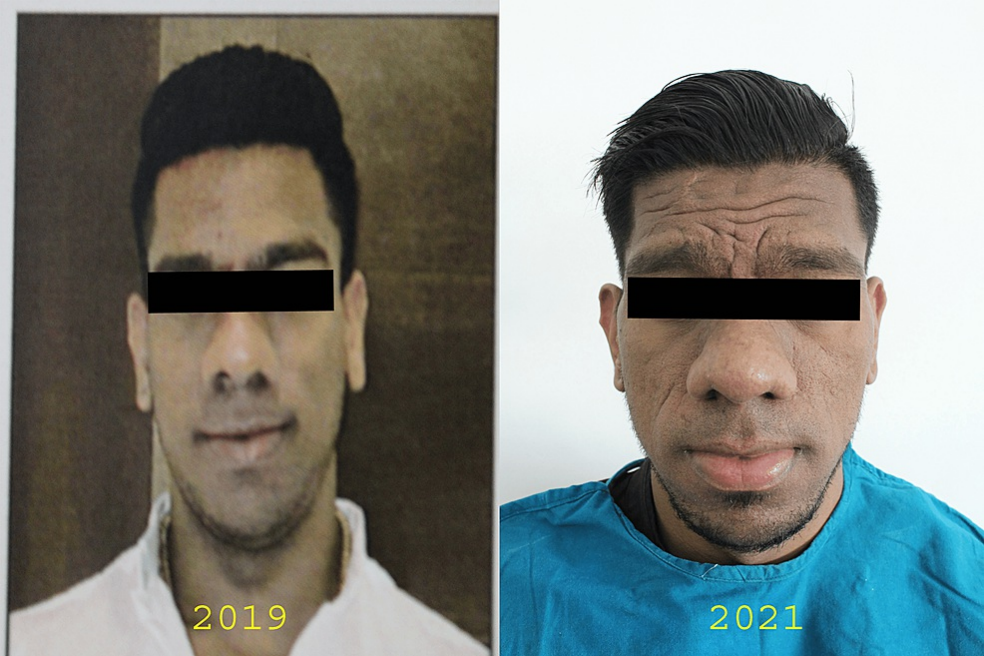
A comparison of photographs showing persistent growth of the mandible

He was started on conservative therapy, which included the use of full-coverage stabilization splints, physical therapy, a soft diet, and muscle relaxants. The pain was evaluated based on a modified visual analog scale. Serial photographs and dental models showed persistent growth of the mandible (Figure 5).

**Figure 6:**
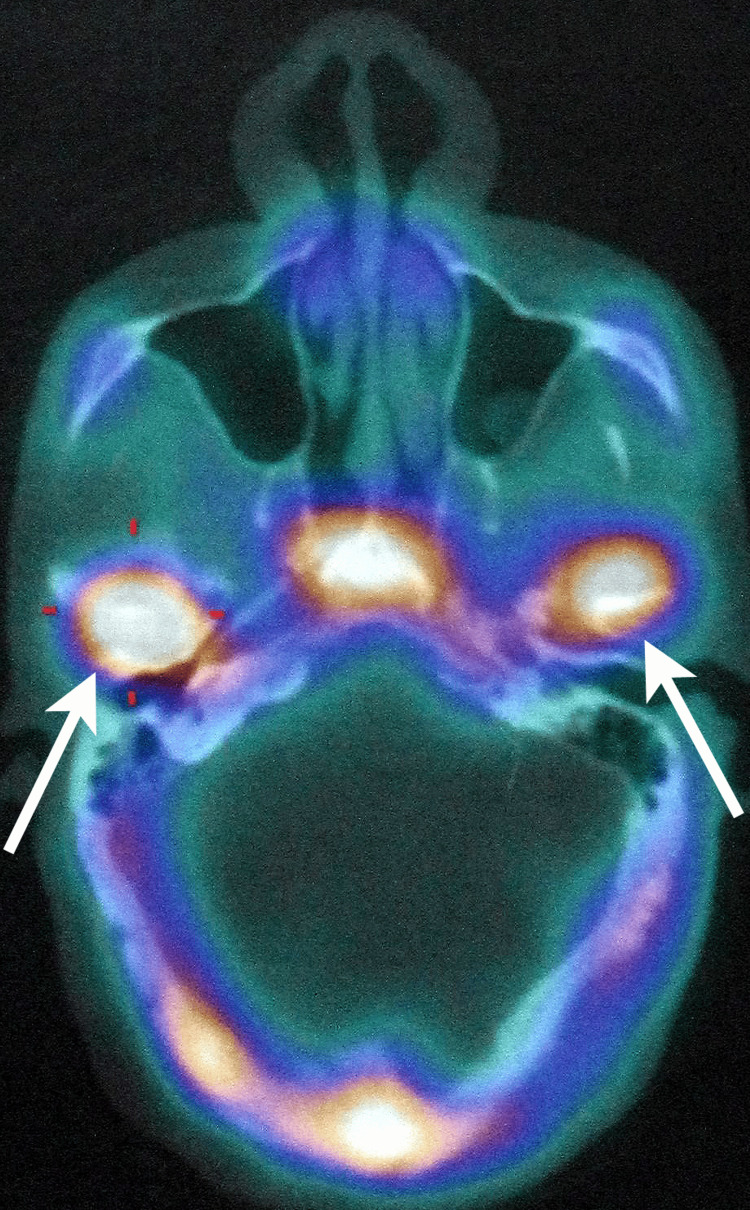
Axial section of Tc-99m SPECT scan showing significantly increased tracer uptake in both condyles (white arrow), suggestive of active bilateral condylar hyperplasia Tracer uptake was significantly high in the right mandibular condyle

Despite eight weeks of conservative therapy, the patient showed no reduction in the intensity of pain in the TMJ region. Technetium-99m bone scintigraphy was done, which showed increased tracer uptake in both condyles, suggestive of active bilateral condylar hyperplasia (Figure 6).

**Figure 7:**
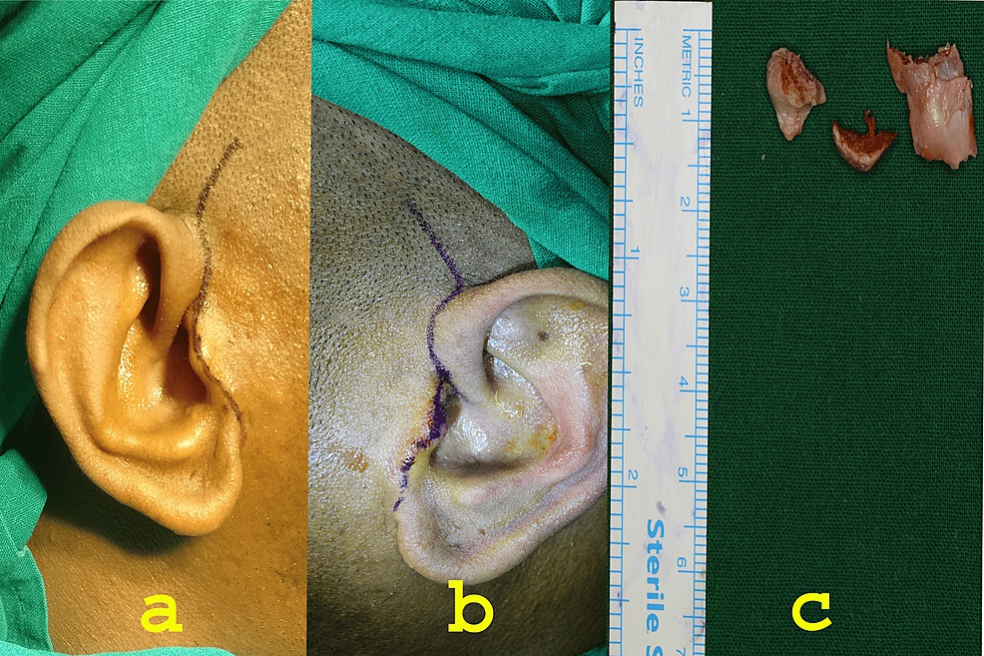
(a) Right preauricular incision; (b) left preauricular incision; (c) resected specimens of condyle

After a thorough systemic evaluation and joint consultation with the neurosurgeon and endocrinologist, the patient was scheduled for bilateral high condylectomy. The patient’s informed consent was obtained for treatment. As a protocol of our institution, consent was also obtained for the use of data, such as photographs, scans, and study models for publication and academic purposes. Standard preauricular incisions were placed, and 5 mm of the superior aspect of the right and left mandibular condyles were excised (Figure 7).

**Figure 8:**
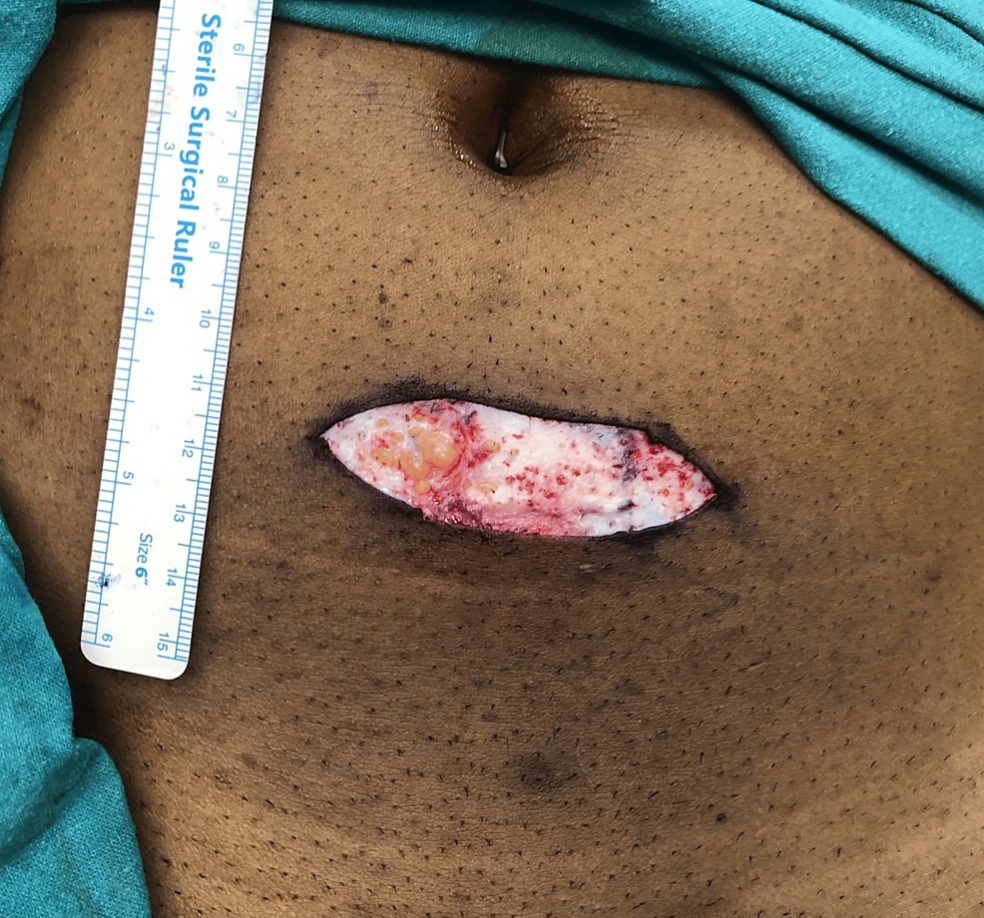
Harvesting abdominal dermis fat through an elliptical incision in the sub-umbilical region Tight closure of the TMJ capsule was achieved

There was noticeable thinning of the right and left articular disc with a perforation at the medial aspect of the right articular disc, which was not noticeable in the MRI. Deformation and perforation of the right articular disc impelled its removal. The abdominal dermis fat graft was harvested from the sub-umbilical region and interposed in the right TMJ (Figure 8).

**Figure 9:**
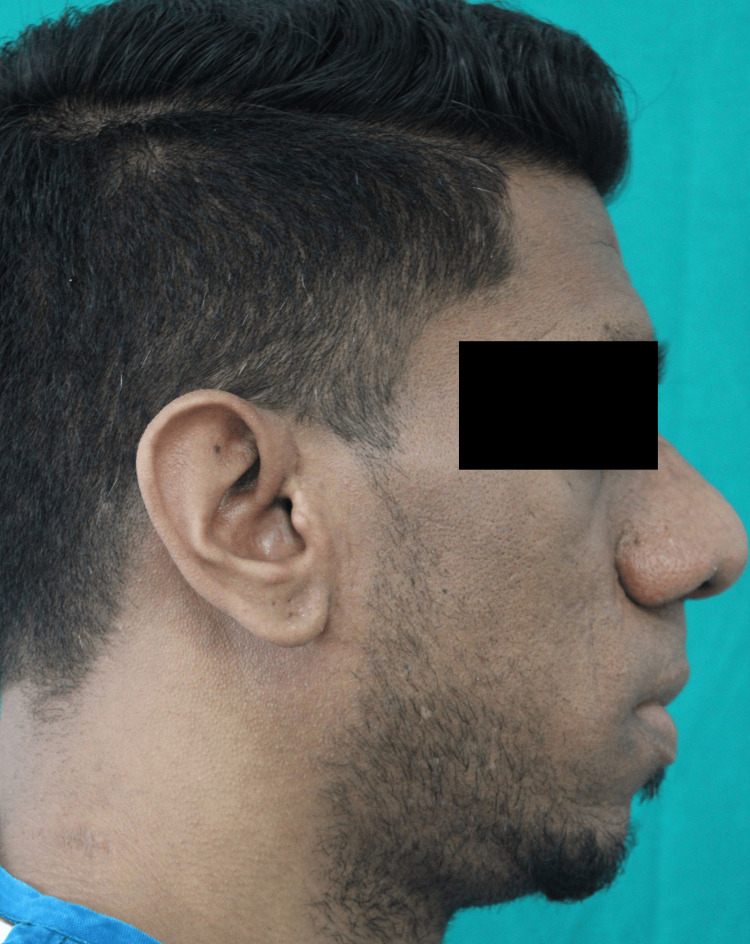
One-year postoperative profile view

The follow-up period showed a marginal decrease in pain intensity. Tenderness over TMJ was significantly reduced two weeks postoperatively. However, pain over the masseter, temporalis region, and headache persisted. The patient was referred to the pain clinic for further management. His six-month postoperative bone scintigraphy showed a marked decrease in the tracer uptake. One-year follow-up photographs and study models showed arrested growth of the mandible (Figure 9).

Because of the persistent increase in IGF-1, the patient was subjected to radiation therapy, which resulted in some reduction in IGF and GH levels. The patient is presently being given a combination of somatostatin analog (octreotide) and dopamine antagonist (cabergoline) to achieve normal levels of IGF-1. There is a definite improvement in his comorbidities following a combination of pharmacological and radiation therapy, which has improved his quality of life. His present IGF-1 and GH levels are still elevated, but there is an overall improvement in sleep pattern and reduced constitutional symptoms like fatigue and myalgia.

## Discussion

The management of morbidities associated with acromegaly essentially requires a multidisciplinary approach. Maxillofacial changes are the second most common findings after acral enlargement [3]. Egyedi suggested a possible correlation between serum GHs and condylar hyperplasia [4]. Muller has reported a case of condylar hyperplasia in a proven case of acromegaly [5]. Obwegeser and Luder observed that the growth stimulation and pattern of growth in acromegaly induced condylar hyperplasia are not the same as the other types [6].

In the report described here, the patient’s primary complaint was severe progressive pain in bilateral TMJ, masseteric, and temporal region, management of which was attempted by conservative measures. However, the failure of conservative measures impelled further investigation, such as MRI and bone scintigraphy. Scintigraphy studies showed increased tracer uptake in both mandibular condyles and sacroiliac joints. High condylectomy is not routinely advocated as a treatment for acromegaly induced condylar hyperplasia since the growth gets arrested after successful management of pituitary adenoma. However, the present case was refractory to surgical and medical management. There was a persistent rise in hormonal levels, resulting in persistent growth of the mandible and continuous prolonged pain. Although the main objective of high condylectomy in the present case was to arrest progressive growth of the mandible, we expected a certain amount of pain relief after surgery due to a reduction in joint overload. Unfortunately, postoperative reduction in pain was not significant. 

High condylectomy has been proven to be an effective surgical modality to arrest the growth of the mandible in the case of condylar hyperplasia [6]. However, its role in acromegaly induced condylar hyperplasia has not been studied much. Serial photographs, cephalograms, and dental models of this patient have revealed that there was no further growth of the mandible in a two-year follow-up after high condylectomy. Incidentally, there has been some reduction in hormonal levels following medications and radiation therapy. So, it may be prejudiced to attribute the arrest of growth of the mandible to high condylectomy alone. The authors do not recommend high condylectomy as a norm to treat all acromegaly induced condylar hyperplasia. Surgical intervention for TMJ should be chosen judiciously, only if there are degenerative changes in TMJ or if a highly disproportionate growth of the mandible poses challenges for secondary skeletal corrections. Also, in case of severe degenerative changes of TMJ, total joint replacement may be considered. Although there was some reduction in TMJ pain, the myogenous pain and headache persisted. TMJ surgery in such cases, purely for pain control, may be unwarranted. Wechsler described multimodal physical therapy and neuromuscular re-education for pain management in a similar case of acromegaly induced facial pain [7].

In the present case, deformation and perforation of the right articular disc necessitated its removal. The role of abdominal dermis fat as a replacement for the disc in TMJ is well established [8]. The MRI done at a two-year follow-up has shown considerable resorption of the grafted fat, which is contradictory to the literature [9,10]. Unfortunately, there was no MRI done immediately following surgery, which could have helped in the quantitative evaluation of the volumetric change in the grafted fat. Despite this resorption, the patient showed improvement in TMJ function.

## Conclusions

Dentists, maxillofacial surgeons, and head and neck surgeons can immensely contribute to the early diagnosis and treatment of morbidities associated with acromegaly till definitive management is done. TMJ high condylectomy should be judiciously used as a treatment modality in acromegaly induced condylar hyperplasia. In those cases, with signs of degenerative arthropathies, it may provide an additional benefit of minimizing these degenerative changes. Surgical treatment of TMJ has little role in the management of facial pain in patients with acromegaly. It may be prudent to add arthroscopy as a diagnostic and treatment modality for early cases of internal derangement. TMJ surgery may be selectively used for refractory cases of acromegaly and those requiring discectomy or total joint replacement.
